# Cultivation of *Heligmosomoides Polygyrus:* An Immunomodulatory Nematode Parasite and its Secreted Products

**DOI:** 10.3791/52412

**Published:** 2015-04-06

**Authors:** Chris J. C. Johnston, Elaine Robertson, Yvonne Harcus, John R. Grainger, Gillian Coakley, Danielle J. Smyth, Henry J. McSorley, Rick Maizels

**Affiliations:** ^1^Institute of Immunology and Infection Research, University of Edinburgh; ^2^Manchester Collaborative Centre for Inflammation Research

**Keywords:** Immunology, Issue 98, *Heligmosomoides polygyrus*, Helminth, Life Cycle, Excretory-Secretory Products, Immunology, Infection, Mouse

## Abstract

*Heligmosomoides polygyrus *(formerly known as *Nematospiroides dubius, *and also referred to by some as *H. bakeri*) is a gastrointestinal helminth that employs multiple immunomodulatory mechanisms to establish chronic infection in mice and closely resembles prevalent human helminth infections. *H. polygyrus *has been studied extensively in the field of helminth-derived immune regulation and has been found to potently suppress experimental models of allergy and autoimmunity (both with active infection and isolated secreted products). The protocol described in this paper outlines management of the *H. polygyrus* life cycle for consistent production of L3 larvae, recovery of adult parasites, and collection of their excretory-secretory products (HES).

**Figure:**
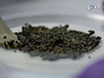


## Introduction

*Heligmosomoides polygyrus* is a natural murine gastrointestinal helminth that is closely related to highly-prevalent human nematode parasites ^1^. In contrast to other nematode models such as *Nippostrongylus brasiliensis*, *H. polygyrus *consistently establishes chronic infection in mice as a direct result of multiple powerful immunomodulatory mechanisms it employs to suppress the host immune response ^2^.

*H. polygyrus *has a direct life cycle: infective L3 larvae are ingested by feco-oral transmission (or administered by oral gavage in the laboratory setting), whereupon they migrate to the subserosal layer of the duodenum and encyst before returning to the intestinal lumen as adult worms approximately eight days after initial infection. Mating and egg production occurs by day 10 and it is possible to harvest adult worms for culture and collection of excretory-secretory products from day 14 onwards ^3^. *H. polygyrus* also interacts with the commensal microflora, with increased Lactobacilli present in infected susceptible mice ^4-6^, and increased levels of* H. polygyrus *infection following exposure of mice to Lactobacilli ^6^.

Active infection with *H. polygyrus *has been shown to protect against immunopathology in many animal models of autoimmunity ^7-10^, colitis ^11,12^ and allergy ^13-16^. There has consequently been great interest in the potential of Excretory-Secretory molecules from this parasite (“HES”) to down-modulate pathology in vivo ^17,18^. Indeed, protective effects are seen following treatment of mice with HES products ^19^ through pathways which are now being identified ^18,20^. Here we describe a protocol for the reliable production of *Heligmosomoides polygyrus *and recovery of its secreted products that can be further utilized for a range of functional biochemical and immunological investigations.

## Protocol

NOTE: All procedures in this protocol are performed in accordance with guidelines set out by the United Kingdom Home Office and the University of Edinburgh Veterinary Services.

### 1. Infection of Mice by Gavage

1. Store *Heligmosomoides polygyrus *L3 larvae in distilled water for up to six months at 4 °C.
2. Before use, wash L3 larvae three times in distilled water: centrifuge at 300 x g for 10 min (with brake), remove all but 500 μl of water (to avoid disturbing pelleted L3 larvae) and resuspend the pellet each time.
3. For the third wash, add water to an exact volume (typically 40 ml) and aspirate 20 µl with a 200 µl tip cut to widen its aperture. Place two 20 µl samples on the surface of a 60 mm culture dish and count the L3 larvae (usually mobile, and best viewed under 50X magnification with a dissecting microscope). Resuspend the final pellet in distilled water to a concentration of 2,000 L3 larvae per ml.
4. For life cycle production, infect 8 week-old F1 (C57BL/6xCBA) mice with 400 *H. polygyrus* L3 larvae in 200 μl of distilled water by oral gavage (restrain mice in the upright position by the scruff of the neck and gently pass the blunt gavage needle through the mouth and esophagus into the stomach). Agitate thoroughly prior to each infection (larvae settle quickly in water) and aspirate 200 µl in a 1 ml syringe; use a dedicated gavage needle with a rounded end. For experimental infection of younger mice (6-8 weeks old), or other inbred strains (*e.g.* C57BL/6 or BALBc), infect mice with 200 L3 larvae.

### 2. Propagation and Maintenance of *H. polygyrus*

1. Place charcoal in the center of a large plastic tub and allow cold tap water to run over it for a minimum of 30 min (unwashed activated charcoal is toxic to L3 larvae). Drain the water from the tub and place the charcoal on two layers of absorbent paper, leaving it exposed to room air until completely dry.
2. If eggs are required, scrape feces first out of the colon with forceps (and scissors if necessary). If a large number of L3 larvae is needed, place mice on a wire grid and collect fecal pellets over several days.
3. Mix the feces with granulated charcoal at a ratio of at least a 1:1, to achieve a consistency just damp enough to adhere to filter paper. Smear a thin layer on the center of some dampened filter paper in a petri dish and place this in a humid box (add some damp paper towel and a dish of water) in the dark for 12-14 days.
4. Remove L3 larvae from day 7 onwards, and collect them on at least two occasions before the paper is discarded. The larvae form a ring around the edge of the filter paper; lift the filter paper out of the petri dish and rinse the larvae that are left of the plate (using a pipette and 5 ml of sterile water per plate) into a 50 ml tube.
5. Lift off the filter paper and harvest the remaining larvae left on the plate with distilled water into a 50 ml tube. Centrifuge the effluent solution at 300 x g for 10 min. Wash the larvae three times with distilled water and then store at 4 °C in up to 50 ml of distilled water until required. NOTE: Larvae remain viable and infective for at least 6 months.

### 3. Collection of Adult *H. polygyrus* Worms

1. Prepare the modified Baermann Apparatus in advance as shown in **Figure 1**.
2. Cull mice fourteen days after infection.
3. Wash the abdomen with 70% ethanol. Cut the skin over the abdomen and pull back to reveal the anterior abdominal wall. Make midline incision to enter the peritoneal cavity.
4. Remove the entire small and large intestine (from proximal duodenum to distal rectum). Place into a dry Petri dish.
5. Straighten the gut along its entire length; excise the feces-containing colon; place this into a separate dish for egg preparations later.
6. Excise the proximal 20 cm of small intestine that contains the adult worms –identified by the relatively thick wall of the duodenum and often a red appearance due to the intra-luminal worms. Place into a (100 mm diameter) Petri dish with 5 ml of Hanks’ Solution, warmed to 37 °C (two specimens per dish).
7. Open the worm-filled proximal gut portion longitudinally with scissors (round-ended scissors are best for this), and scrape down inside of gut lining with two glass slides to remove the worms. Then discard the clean gut wall.
8. Tip worms into small muslin bags, staple closed and secure with paperclips around the edge of the glass funnel (**Figure 1**).
9. Fill funnel with Hanks’ Solution and add approximately 4 Petri dishes of worms into each funnel.
10. Place apparatus in 37 °C incubator for 1-2 hr, gently agitating half way through to dislodge debris from the gut preparation that may occlude the muslin filter. Take care to avoid spillage of debris outside the muslin bag – this will cause contamination of the final HES preparation. Adult worms should have slowly migrated through the muslin cloth and settled at the bottom of the glass test tube. Carefully detach the test tube from the connecting rubber hose over the sink (taking care to avoid losing worms at this point).
11. Using a plastic pipette, transfer the worms into a 50 ml tube and wash six times with Hanks’ Solution (allow worms to settle with gravity, remove media with a stripette, add 40 ml of Hanks’ Solution and repeat five times). NOTE: worm culture must be kept sterile from this point onwards.
  1. Move to a laminar flow hood and wash another six times in sterile Hanks’ Solution supplemented with 100 U/ml penicillin and 100 µg/ml streptomycin, ready for *in vitro* culture.
12. Count the adult worms recovered by taking two samples of 20 µl taken up with a yellow tip cut to widen its aperture; expect approximately 50% of the quantity of inoculated larvae.

### 4. Setting Up Cultures for HES: Medium Preparation, Washing Adult Worms

1. Soak the worms from 3.11 in approximately 10 ml of RPMI supplemented with 10% Gentamycin for 20 min, leaving the tube resting at an angle to ensure worms are fully covered.
2. Perform this in a laminar flow hood: wash again six times with Hanks' Solution (supplemented with 5 U/ml penicillin and 5 μg/ml streptomycin).
3. Prepare *H. polygrus* media.
4. Maintaining sterility in a laminar flow hood, to 500 ml of RPMI1640, add 11.1 ml of 45% glucose (final concentration 1.2% as RPMI1640 contains 0.2% glucose), 5 ml of 100x Peniclllin-Streptomycin (final concentration 5 U/ml penicillin, 5 μg/ml streptomycin), 5 ml of L-glutamine (final concentration 2 mM), and 5 ml of Gentamycin (final concentration 1%). Do not add FCS.
5. Aliquot worms into vented T25 flasks, approx. 1,000 worms in 15 ml *H. polygyrus* media per flask, and place upright in 37 °C incubator (5% CO_2_) for 3 weeks.

### 5. Preparation of HES

1. Collect HES-containing culture media from cultures at intervals of no longer than twice per week, setting aside the first collection after 24 hr of culture (due to high levels of LPS contamination and host proteins – can be processed separately or discarded). Keep each collection separate and clearly labeled with the date and batch number. Replace with an equal volume of *H. polygyrus* media on each occasion.
2. Centrifuge HES-containing media at 400 x *g* for 5 min. Then filter sterilize through 0.2 µm low-protein-binding filters into 50 ml tubes. Store in the -20 °C freezer clearly labeled with date of worm harvest and date of HES collection.
3. After 21 days of HES collection from culture, discard worms.
4. Pool 500-1,000 ml of HES supernatant (usually from frozen stock, and not including the first 24 hr collection) and concentrate over a 3,000 MWCO filter in the ultrafiltration device under nitrogen pressure. NOTE: Be very careful not to let the filter run dry.
  1. To set up the filter device, first wash the 3 kDa membrane shiny side down in a 1 liter beaker with distilled water for 3 x 20 min whilst stirring. Assemble the ultrafiltration device as per manufacturer’s instructions with filter membrane shiny side up. Place in cabinet at 4 °C and pass 50 ml of distilled water through before starting to concentrate pooled HES.
  2. Add each tube of HES into the filtration device as required (100-140 ml per day), until the volume is concentrated down to 2-5 ml.
5. In order to remove contaminants from the HES-containing culture media, add 50 ml of pyrogen-free PBS to the filtration device and and then concentrate down to approximately 2 ml. Repeat this step twice (150 ml of PBS in total). Transfer HES into a 15 ml tube, filter sterilize (with a 0.2 μm filter) in a laminar flow hood and measure protein concentration using a spectrophotometer (E_280_ = 10) or by Bradford assay.
6. Aliquot, label with batch number and date, and freeze at -80 °C.
7. Perform a chromogenic LAL assay according to the manufacturer’s protocols on each batch of HES prior to use. If LPS levels are greater than 1 U LPS per 1 µg protein, consider not using this batch for *in vivo* experiments or *in vitro* cultures.
8. Process the HES collected at 24 hr separately in the same manner; it will contain LPS and some host proteins and, while not suitable for functional experiments, it is a useful source of individual molecules that may be isolated by monoclonal antibody affinity purification.

## Representative Results

Susceptibility to infection with *H. polygyrus* is controlled to a large degree by the genetic background of the mouse strain (**Table 1**); C57BL/6 and CBA mice are highly susceptible ^21,22^. For maintenance of the parasite life cycle, the F1 hybrid between these two strains has been chosen for its ability to withstand much higher worm burdens without morbidity (excessive intestinal epithelial damage) compared to either parental strain. Oral gavage of 400 L3 larvae is used to maintain the life cycle in F1 mice (resulting in adult worm burdens shown in **Figure 2**), whilst a dose of 200 L3 larvae is generally used for experiments in homozygous inbred strains (*e.g.*, C57BL/6 or BALBc). However, this dosage may need to be reduced depending on environmental co-factors that may differ between animal facilities, such as variations in gut flora.

Batches of HES have proven reproducible efficacy in functional assays and in protein composition; moreover, when supernatants from each successive week of culture were analysed, up to a total of 4 weeks, the protein profiles were found to be very similar (**Figure 3**)**. **When HES concentration is measured by Bradford assay (see **5.5**), the total protein is usually approximately 1 mg/ml (**Figure 4**).

An alternative method of concentrating HES is with a centrifugal concentrator (eg Vivaspin 3-kDa cut-off membrane), in which samples are spun at up to 4,000 g in a swing-out rotor centrifuge, removing buffer salts and low molecular weight components. Centrifugal concentration is best suited to small processing volumes (1-10 ml) and are limited to a maximum concentration factor of approximately 30x.

When collecting HES, avoidance of contamination is critical. To avoid contamination with host molecules, we discard HES-containing culture media from the first 24 hr after adult worm harvest from the mouse intestine. We also quantify the level of LPS contamination in each batch of HES with a Chromogenic LAL assay (see 5.7). 1 U of LPS equates to ~100 pg LPS and levels below this are considered negligible ^23^. In our hands, most batches of HES are significantly below this limit, the mean concentration of LPS in HES being 0.23 U/µg (**Figure 5**). The effects of LPS in *in vivo* models of pathology (for instance the suppression of asthmatic responses) requires at least 10 ng of LPS ^23^. Hence *in vivo* administration of 5 µg HES from a batch with 100 pg LPS/µg HES will include 500 pg LPS, well below the effective concentration where LPS becomes a problem.


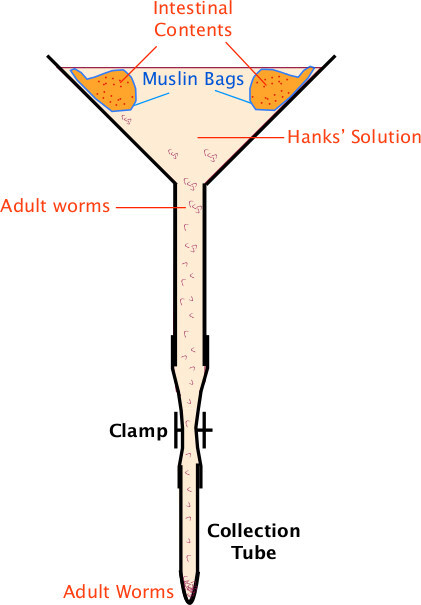
**Figure 1: Baermann Apparatus **Baermann Apparatus setup for collection of adult *H. polygyrus* (as described in section 2).


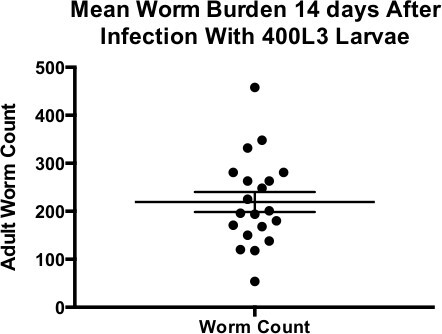
**Figure 2: Mean Worm Burden 14 Days After Infection with 400 L3 Larvae **Mean worm burdens 14 days after infection with 400 L3 larvae. Data points shown are from 19 separate rounds of infection of C57BL/6xCBA mice. Mean ± SEM shown.


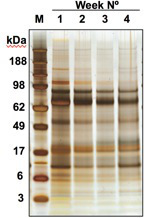
**Figure 3: Protein Profiles of HES From Successive Weeks in Culture **SDS-PAGE profile of HES proteins collected in successive weeks of culture.


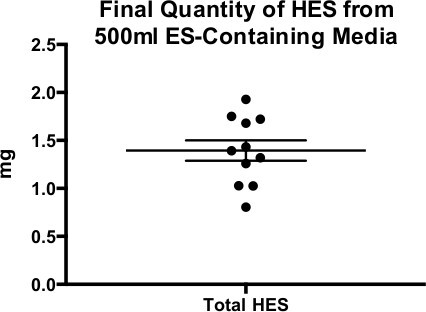
**Figure 4: Final Quantity of HES from 500 ml ES-Containing Media **Yield of HES protein from 11 different batches derived from approximately 500 ml of culture supernatant. Mean ± SEM shown.


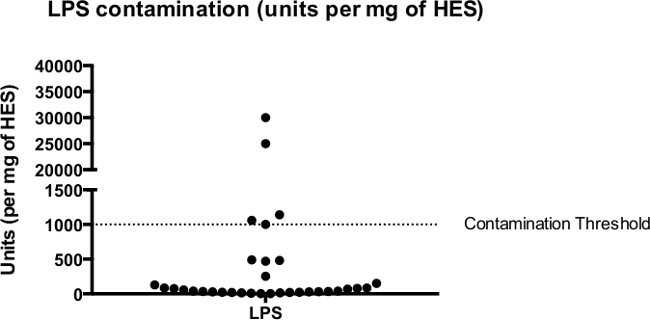
**Figure 5: LPS Contamination of HES **Levels of LPS contamination in 41 batches of HES measured by the Limulus ameobocyte assay. Median LPS concentration = 86 U per mg of HES.


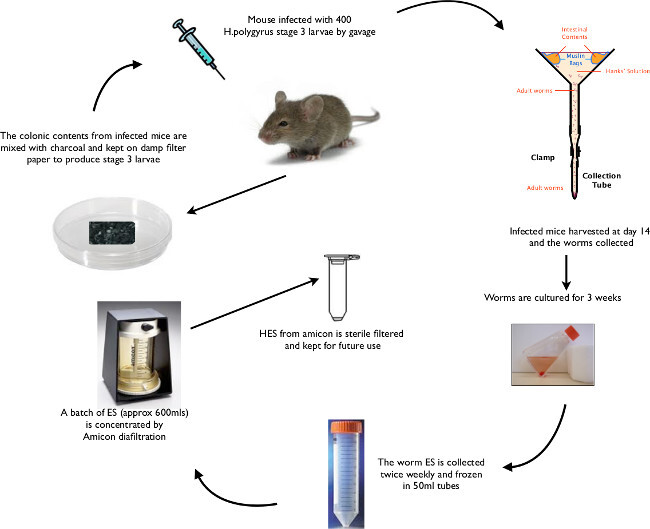
**Figure 6: Animated Schematic of *****H. polygyrus***** Life Cycle **Summary of key life cycle stages from oral gavage of L3 larvae, through recovery of larvae and adult worms to isolation of HES.


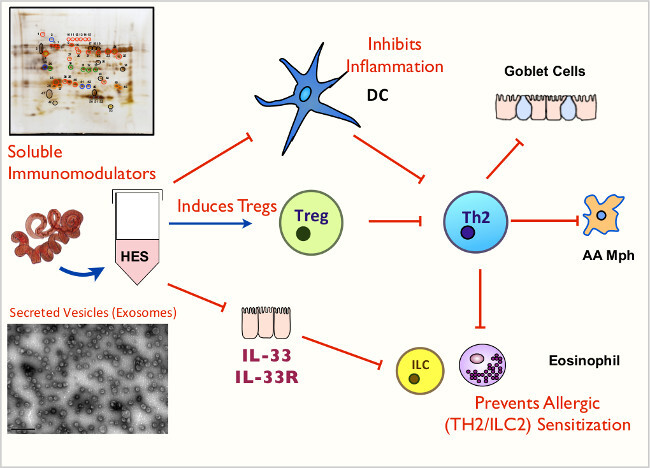
**Figure 7: Animated Schematic of HES and its Functions **Key immunomodulatory effects of soluble mediators and exosomes contained within HES.

**Table:** 

**Genotype (and background strain)**	**Primary Infection Phenotype**	Reference
		
Inbred strains		
A/J, CBA, C3H	Highly susceptible	22,29
C57BL/6 and C57BL/10	Susceptible	22,30
BALB/c, DBA/2, 129/J	Intermediate	22,31
NIH, SJL, SWR	Low susceptibility	22,32
		
**Transgenic for cytokines or cytokine receptors**	
IL-1β^-/-^	More susceptible	33
IL-1R-/-	Less susceptible (a); no change in susceptibility but increased granulomas (b)	(a) ^33^
(b) ^34^
IL-2Rβ Transgenic (C57BL/6)	Resistant	35
IL-4-/-	Higher fecundity	36
IL-4R-/- (C57BL/6 or BALB/c)	Highly susceptible	22
IL-6^–/–^ (BALB/c or C57BL/6)	Highly Resistant	37
IL-9 Transgenic (FVB)	Resistant	38
IL-21R^ –/–^ (C57BL/6)	Deficient Th2, decreased granuloma gormation	39,40
IL-25-/- (BALB/c)	More susceptible	33
IL-33R (T1/ST2) -/- (BALB/c)	No change in susceptibility	33
TGFβRIIdn (C57BL/6)	High Th1, Increased susceptibility	41,42
		
**Transgenic for T cell markers**	
CD28–/– (BALB/c)	Marginally higher fecundity	43
CD86 (B7-2)–/– (BALB/c)	Higher fecundity	44
OX40L–/– (BALB/c)	Higher fecundity	45
		
**Transgenic for innate Immune loci **	
Type 1 interferon receptor (IFNAR)-/- (C57BL/6)	Higher fecundity, more granulomas	34
MyD88–/– (C57BL/6)	More resistant, more granulomas	34
C-KitW/Wv (WBB6)	More susceptible	46,47

**Table 1: Primary infections with** ***H. polygyrus*** **in genetically different and gene-targetted mouse strains.**

## Discussion

The life cycle of *H. polygyrus *proceeds in a reliably consistent fashion. Following natural ingestion or oral gavage of L3 larvae on day 0, cysts begin to form under the duodenal serosa by day 5, progressing to larval moults and then emerging as adult worms into the gut lumen from day 10, eggs can be seen in feces from day 14 and granulomas are visible on the duodenal serosal surface from day 21. The protocol described above (and summarized in **Figure 6**) allows for high-throughput production of *H. polygyrus* excretory-secretory products (HES), in addition to reliable recovery of viable L3 larvae for future experimental and life cycle infections.

*H. polygyrus* infection has been shown to be protective in models of asthma dependent on OVA or Der p 1 (House dust mite allergen)^14^. Furthermore, suppression of airway inflammation could be transferred with CD4^+^CD25^+^ regulatory T cells ^14^ or CD19^+^CD23^hi ^regulatory B cells ^24^ from non-sensitised, *H. polygyrus*-infected mice. In models of autoimmunity, *H. polygyrus *infection has been shown to be suppressive in the experimental autoimmune encephalomyelitis (EAE) model of multiple sclerosis ^9^, and suppression can be transferred with either CD4+ T cells or CD19+ B cells from infected mice ^24^.

*Heligmosomoides polygyrus* excretory-secretory products (HES) modulate the host immune response and suppress Th2-mediated inflammation by a number of mechanisms (outlined in **Figure 7**), including: a) Inhibition of dendritic cell responsiveness to stimulation ^25^, b) induction of CD4^+^Foxp3^+^ regulatory T cells ^18 ^. and c) blockade of IL-33 production ^20^. HES has been shown to be protective when administered at sensitization or challenge in the OVA-alum model of asthma ^19^ and also when administered intranasally with Alternaria extract allergen ^20^, through suppression of early IL-33 release. Avoidance of LPS contamination of HES is often crucial for the success of future immunological experiments. In the protocol outlined here, the critical steps in achieving this are to ensure that debris contained within the muslin bags of the Baermann Apparatus does not enter the final HES preparation (see 2.10) and to set aside ES solution from the first 24 hr of culture (see 5.1).

Over recent years, HES has been thoroughly characterized at the proteomic level with over 370 distinct proteins identified ^26,27^. In addition, ES from the 4th stage larvae has been subjected to proteomic analysis ^28^. Ongoing work includes characterizing the major glycan components of HES, known to be strongly immunogenic ^3^, and secreted micro-RNAs that are encapsulated in 50-100 nm vesicles or exosomes (Buck *et al*., submitted for publication). With the establishment of reproducible protocols for the collection of ES from this highly immunomodulatory parasite, and with extensive proteomic data identifying the molecular components of HES, the stage is now set for the identification and therapeutic testing of individual molecules from *H. polygyrus* that can mediate key effects on the host immune system.

## Disclosures

The authors have no conflicts of interest.
